# Development of a BIM-based bridge maintenance system (BMS) for managing defect data

**DOI:** 10.1038/s41598-023-27924-6

**Published:** 2023-01-16

**Authors:** Sai Li, Zhongjian Zhang, Daming Lin, Tongrui Zhang, Lu Han

**Affiliations:** 1 Department of Civil Engineering, School of Engineering and Technology, China University of Geosciences (Beijing), Beijing, 100083 China; 2 Research Institute of Highway Ministry of Transport, Beijing, 100088 China; 3 Huayu Architectural Design Ltd., Shenyang, 110170 China; 4 Xinjiang Production and Construction Corps Highway Science and Technology Co. LTD, Urumqi, 830000 China

**Keywords:** Civil engineering, Software

## Abstract

Bridges might experience many defects during use, such as pavement cracks and reinforcement corrosion, which easily produce an accumulated impact that threatens bridge safety. Thus, there is a need for the regular inspection and maintenance of bridges. This paper presents a bridge maintenance system (BMS) based on building information modelling (BIM), which is utilized in bridge defect information management using a digitalization method. A bridge defect three-dimensional BIM (BIM3D) library is established and combined with a bridge model to visualize bridge defect conditions. Based on bridge inspection data, bridge defect information is digitally classified and encoded according to the international framework for dictionaries (IFD) standard and used to establish a database. An evaluation of bridge technical conditions is performed, and the results are graded and displayed in different colours, reflecting the visualization function of BIM technology. Maintenance suggestions are provided according to the range of bridge technical condition scores, reflecting the informatization function of BIM technology. With the Xinjiang Cocodala Bridge in China as a case study, a bridge BIM3D model and inspection data are imported into the BMS to utilize the functions of ‘visualization of bridge defect conditions’, ‘evaluation of bridge technical conditions’ and ‘recommendations of bridge maintenance methods'.

## Introduction

With the rapid development of transportation infrastructure construction, bridge engineering is gradually showing large-scale and multiunit characteristics. An increasing number of bridges have defects such as insufficient bearing capacity and key structural failure under the natural environment and vehicle load circumstances^1^, which bring a great risk to bridge operational safety. The accumulation of defects will seriously affect bridge safety and cause a series of hidden dangers to public safety. Therefore, it is important to carry out regular inspection, evaluation and maintenance work on bridges^2^. However, there appear to be many problems in traditional methods of bridge maintenance patterns, such as difficulty in managing a large number of defects without a unified standard and displaying bridge conditions. Bridge maintenance systems (BMSs) have been widely developed to assist in the maintenance and management work of bridges with digital solutions. Various countries have developed their BMSs, such as the Preservation, Optimization and Network Information System (PONTIS)^3^ in America, Danish Bridges and Roads (DANBRO)^4^ in Denmark, the National Structures Database (NATS)^5^ in England, the Japanese Bridge Management System (J-BMS)^6^ in Japan, the Seo-Hae Bridge Management System (SHBMS)^7^ in Korea, the China Highway Bridge Management System (CBMS)^8^ in China and BMSs for urban bridges^9^, all of which provide the functions of recording information in the full life cycle of bridges. However, most of the current BMSs utilize a two-dimensional (2D) approach to record and store information without visual display and dynamic integration, which leads to inefficient work for bridge maintenance. In this case, the technical means of building information modelling (BIM) are introduced to significantly compensate for the shortcomings of the existing system^10^. BIM is a three-dimensional (3D) model that contains all the information of engineering projects from the design stage to construction and operation^11^, providing functions of visualization, informatization and integration^12^. Additionally, BIM can share engineering project information with stakeholders for viewing and updating of any code during the life cycle of projects to efficiently assist management^13^. To realize the interoperability of the BIM model, the industry foundation class (IFC) was proposed for data exchange, which is an international BIM open standard^14^. The standard of the international framework for dictionaries (IFD) was used as a tool to encode and transform BIM data in a unified form for interactivity^15^. The IFD method defines all attributes of target objects in a global unique identifier (GUID) manner to eliminate differences between data recording, which improves the accuracy of the model link and data sharing.

To combine BIM technology with BMS, some researchers have focused on establishing structure analysis models and evaluation systems. Dan and Shim^16^ proposed a schematic BIM-based BMS that collaborates with an automated inspection task using an augmented reality (AR) device. Sun et al.^17^ established a structural health monitoring model of the Jiangyin Bridge to carry out maintenance work for the load performance of suspension bridges. Lee et al.^18^ established BIM models of bridge components containing structural parameter information. Deng et al.^19^ developed a bridge structural safety monitoring system based on BIM technology that can release an early warning signal for bridge operation conditions. Shim et al.^20^ proposed a new generation of BMSs by using a digital twin BIM model concept to enhance the decision-making process. Xu et al.^21^ constructed a comprehensive evaluation framework for bridge structural conditions based on visual detection data. Orcesi et al.^22^ added a data evaluation BIM model related to structural health monitoring into the construction cost analysis of bridges to optimize bridge maintenance methods. Li et al.^23^ developed a long-span bridge health condition monitoring system that combines bridge sensor data with an environmental BIM model. The inspection and analysis of structural conditions for bridges are carried out once every one to three years so that the BMS, which is merely aimed at structural safety, can obtain data over a long time interval, which easily neglects defect accumulation from daily use, thus causing serious damage to bridges. However, a few studies have employed BMSs for managing defects by monitoring defect data and bridge technical conditions in real time. Therefore, it is essential to carry out bridge maintenance work by comprehensively combining BIM and defect data.

This study aimed to develop a BIM-based BMS for managing defects, which achieves the targeted deployment of maintenance of daily bridge defects and evaluation of bridge technical conditions. A bridge defect model library is established based on BIM and linked to the bridge components BIM model to display the condition of defects on bridges. A collection workflow for bridge defect data is formulated into a database, and its encoding and classification are completed. Bridge defect conditions are fed to the BMS in real time, and a score of the bridge technical condition is calculated to provide corresponding maintenance suggestions. Taking a case study of the Xinjiang Cocodala Bridge as an example, we import its three-dimensional BIM (BIM3D) model and inspection data into the BMS to realize BIM visualization, informatization and integration functions.

## Methods

### Workflow of the development and utilization of BMS

**Figure 1:**
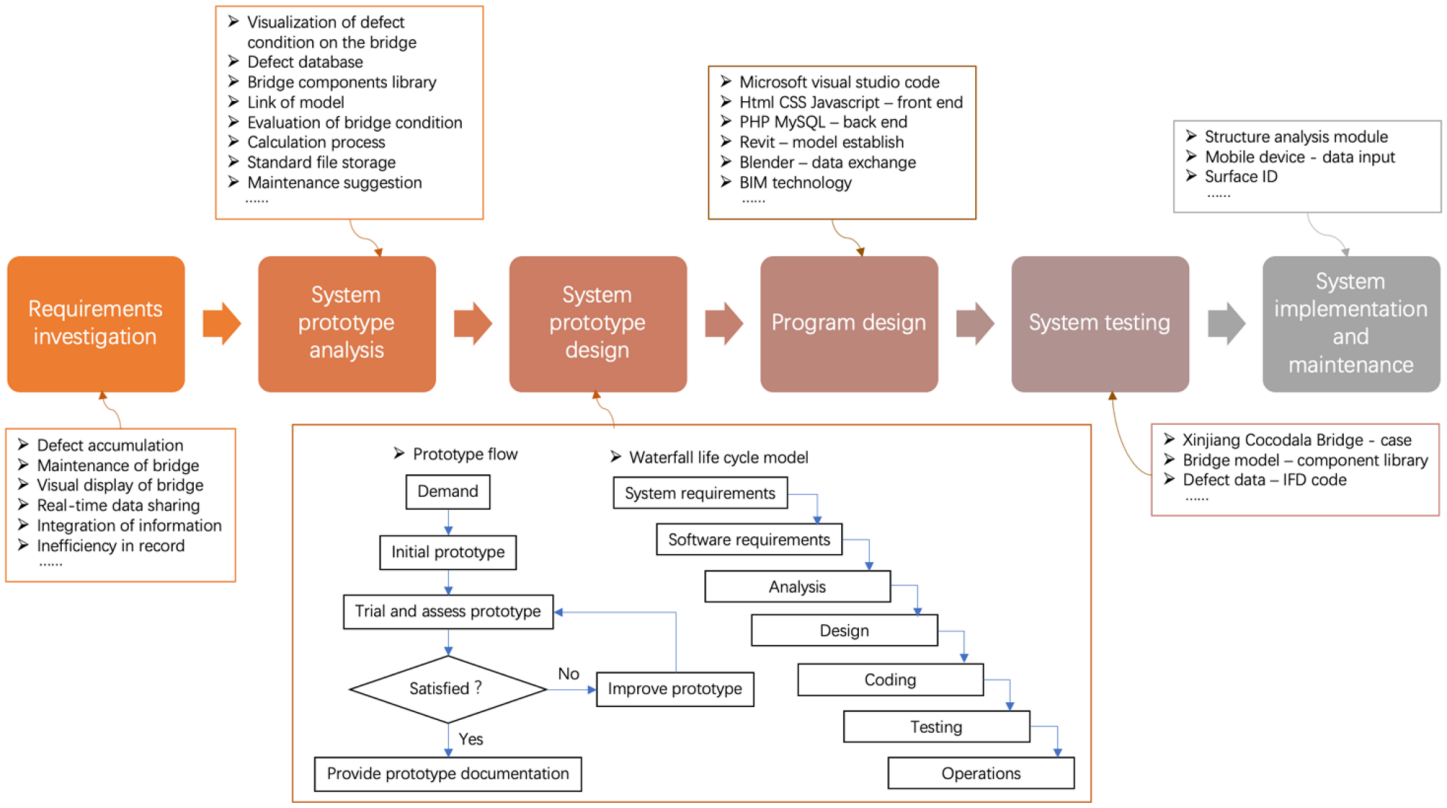
Development flow of the BIM-based bridge maintenance system.

The BMS was developed by the prototyping method and combined with the life cycle development method to visually display the requirements. The development process is divided into six procedures: requirements investigation, system prototype analysis, system prototype design, program design, system testing, system implementation and maintenance. Based on the evolutionary method of prototyping and the waterfall model, the functions of the system are improved in the repeated communication and eventually form a complete system, which is utilized in the front-end activities, data interaction and function module. The BMS was developed as a web-based standalone platform using Microsoft Visual Studio Code tools and several languages such as Hypertext Markup Language (HTML), Cascading Style Sheets (CSS), and JavaScript for the front end and Hypertext Preprocessor (PHP) and Structured Query Language (MySQL) for the back end. The development workflow is shown in Fig. 1.

**Figure 2:**
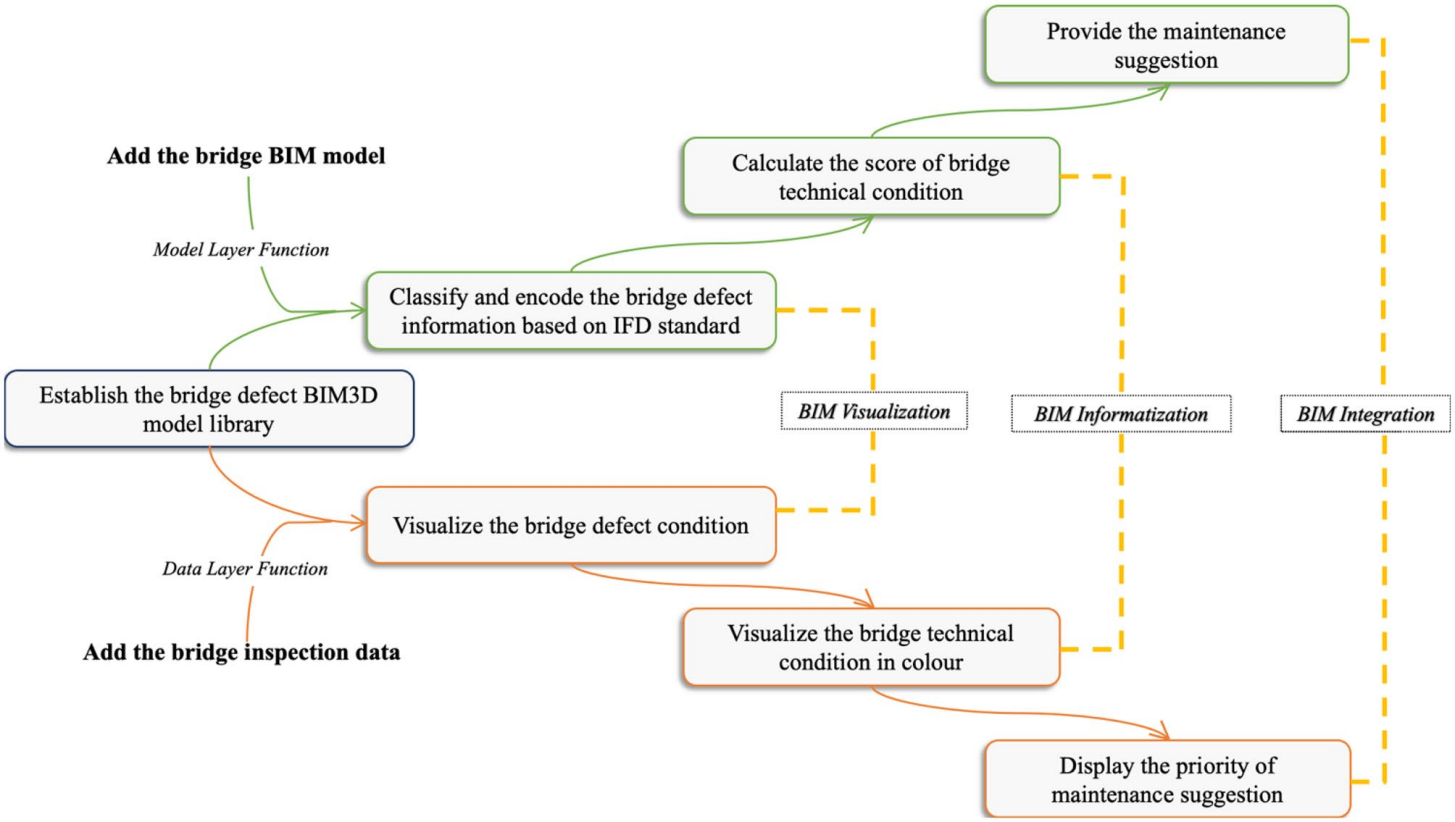
Utilization flowchart of the BIM-based bridge maintenance system.

The BMS realizes the functions of management from the data and model layers, corresponding to the BIM technology visualization, informatization and integration functions. In terms of the function of the data layer, the real bridge inspection data are imported to establish a defect database by encoding according to the IFD standard. Based on the bridge defect database, the technical condition of the bridge is evaluated with a rating score, and maintenance suggestions are provided according to the results. In terms of the function of the model layer, a bridge defect BIM model library is established and combined with the bridge BIM3D model to visually display defect conditions. Different grades of bridge technical conditions are displayed by colour to show the synchronous maintenance status of the bridge. The utilization workflow is shown in Fig. 2. In particular, the system is available for any bridge to utilize the functions after importing the BIM model and the inspection data.

### Establishment of a bridge defect BIM3D model library

The bridge defect BIM model library is categorized into four types to establish the damage model, corrosion model, deficiency model and distortion model. The BIM3D model is designed based on the geometric characteristics of the defects it represents. As shown in Table 1, each type of defect has its own corresponding BIM3D model.

**Table 1:** Classification of bridge defect BIM models.

Category	Type	Geometry Design	Primitive
Damage model	Surface crack	Slender cuboid	
	Surface honeycomb and scale	Cube	
	Surface spalling	Hexagonal cube	
Corrosion model	Concrete corrosion	Triangular cube	
	Reinforcement corrosion	Curved cylinder	
	Cable corrosion		
Deficiency model	Structural member losses	Cylinder	
	Aggregate exposure		
	Structural member ageing and failure	Cone	
Distortion model	Pier deformation	Pentagonal cube	
	Cable	Torus	

### Coding of bridge defect data based on the IFD standard and visualization display of defect conditions

The IFD standard lists the terms and structures that can be used as the basis for a standardized BIM model data catalogue. The coding principles refer to the Classification and Name Code Standard of Railway Engineering Information Model^24^, which provides three categories in BIM national standards, including reference, expansion and independently compiled. Partial encoding categories are summarized in Table 2.

**Table 2:** Bridge defect data classification coding based on the IFD standard.

Number	Name	Compilation principle
10	Architecture classification by function	Expansion of BIM national standards
11	Architecture classification by form	Reference to BIM national standards
12	Architectural space classification by function	Expansion of BIM national standards
13	Architectural space classification by form	Reference to BIM national standards
14	Elements	Reference to BIM national standards
15	Performance	Reference to BIM national standards
22	Professional field	Expansion of BIM national standards
32	Tools	Reference to BIM national standards
33	Participation information	Reference to BIM national standards
40	Material properties	Reference to BIM national standards
41	Attribute characteristics	Reference to BIM national standards
62	Bridge type	Independent compilation
63	Bridge structural components	Independent compilation
64	Bridge operation	Independent compilation
65	Bridge shareholders	Independent compilation
66	Bridge organization	Independent compilation
67	Bridge location	Independent compilation
68	Bridge defect characteristics	Independent compilation
69	Bridge maintenance	Independent compilation

**Figure 3:**
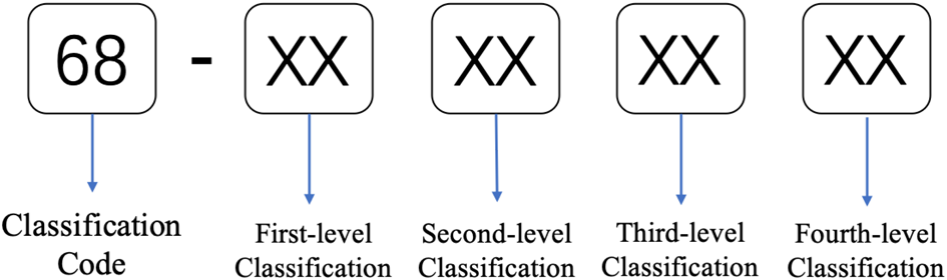
Example format of the code writing principle.

The category of “68-Bridge defect characteristic” is taken as an example to explain the coding process. The general format of the code is called the total number coding method, which consists of the number and objects in the table and is connected by “-”. The length of the code is no more than 15 bits, 2 digits, and is divided into 2 levels, supplemented with “00” when the hierarchy number is insufficient. Figure 3 shows the code writing principle for this example.

The first-level classification is the type of defect, the second-level classification is the detailed defect information, and the third-level and fourth-level classifications are the types subdivided according to defect characteristics. The coding classification of “68 categories” is shown in Table 3, which can be extended and supplemented, such as the fifth level for “crack length” and the sixth level for “crack width”. Moreover, when a defect condition is uploaded to the BMS, the data will be encoded automatically, and the specific ID of the defect is generated with a prefix. For example, if a “transverse crack” defect is uploaded to the system, the ID generated is “1-68-10 10 10”. If another “transverse crack” defect is uploaded again, then the ID generated is “2-68-10 10 10”.

**Table 3:** “68-Bridge defect characteristics” coding classification.

Code	First-level	Second-level	Third-level	Fourth-level
68-10 00 00	Defect type			
68-10 10 00		Cracks		
68-10 10 10			Transverse cracks	
68-10 10 20			Longitudinal cracks	
68-10 10 30			Vertical cracks	
68-10 10 40			Horizontal cracks	
68-10 10 50			Circular cracks	
68-10 10 60			Crocodile cracks	
68-10 10 60 10				Bursting cracks
68-10 20 00		Corrosion		
68-10 20 10			Chemical corrosion	
68-10 20 10 10				Sour corrosion
68-10 20 10 20				Rusting
68-10 20 10 30				Hydrogen embrittlement
68-10 30 00		Defect		
68-10 30 10			Abrasion	
68-10 30 10 10				Surface voids
68-10 30 20			Spalling	
68-10 30 20 10				Block falling
68-10 30 30			Aggregate exposure	

**Figure 4:**
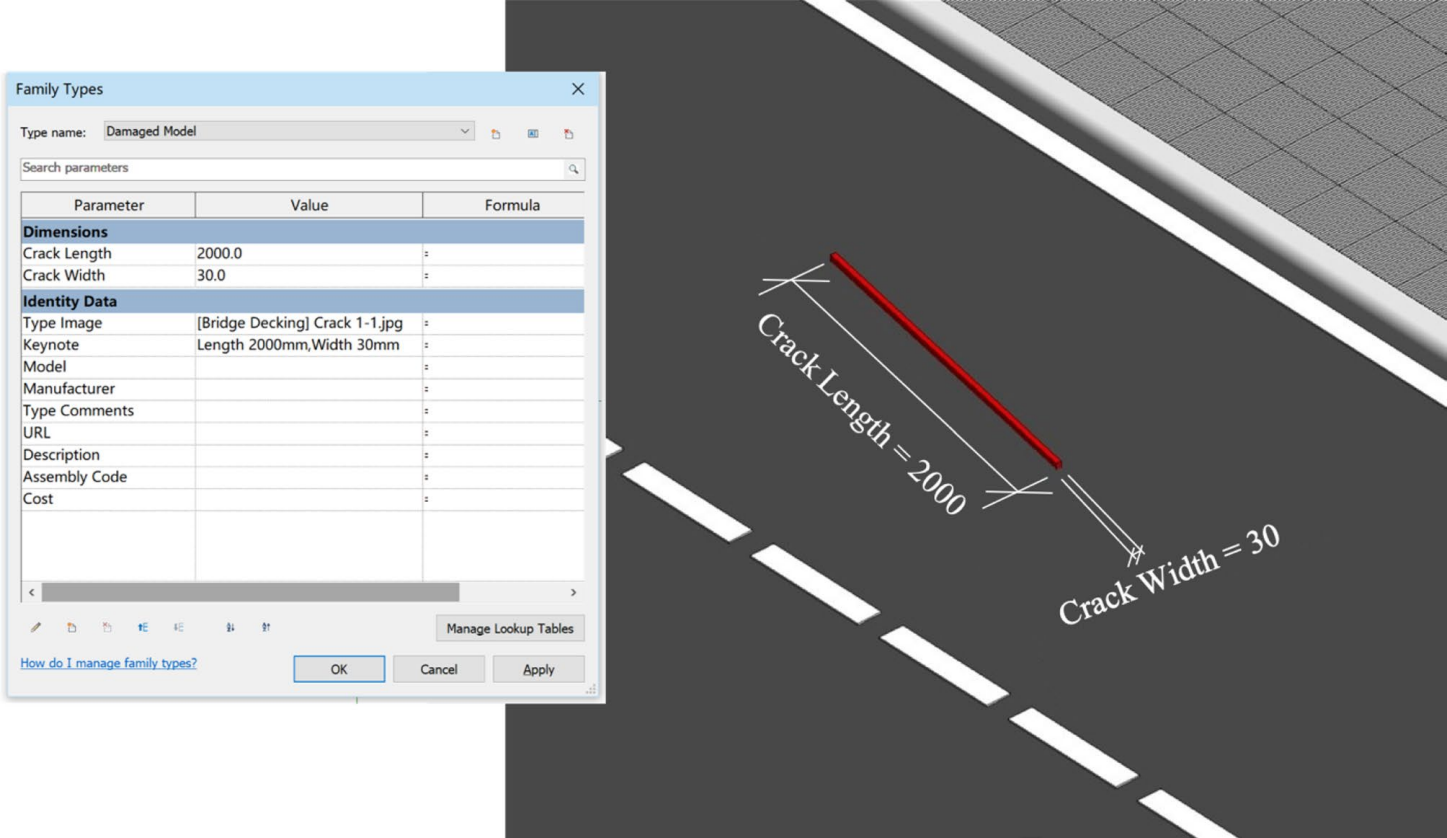
Combination of a concrete crack defect BIM model and bridge BIM model.

The bridge component BIM model is imported to visually display the defect condition by adding the defect model to the bridge model with data exchange and link capabilities(Fig. 4). Based on ISO 19,650-2:2018^25^, the code of bridge model data is mainly divided into three parts of superstructure, substructure, and bridge decking, and their components are further subdivided. To link to defect data, the bridge model’s common data environment (CDE) is established to support the collaborative production of information code and the interactivity of the BIM model.

### Evaluation of the bridge technical condition rating score

**Figure 5:**
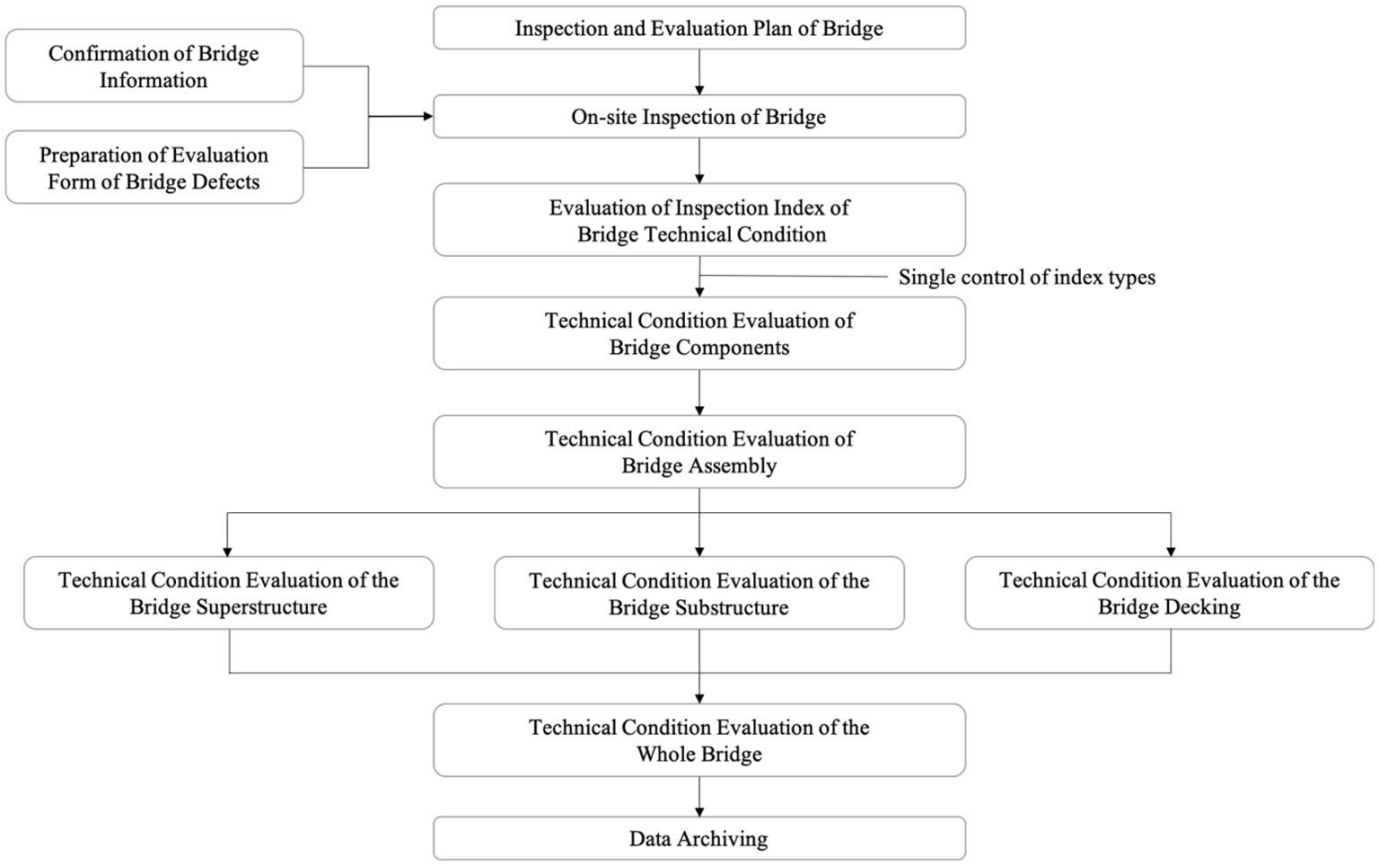
Workflow of bridge technical condition evaluation.

The evaluation score of bridge technical conditions is calculated based on the Standards for Technical Condition Evaluation of Highway Bridges^26^ and adopted with the method of multi-hierarchical comprehensive evaluation and single control of index types. The flowchart of evaluation is shown in Fig. 5.

**Figure 6:**
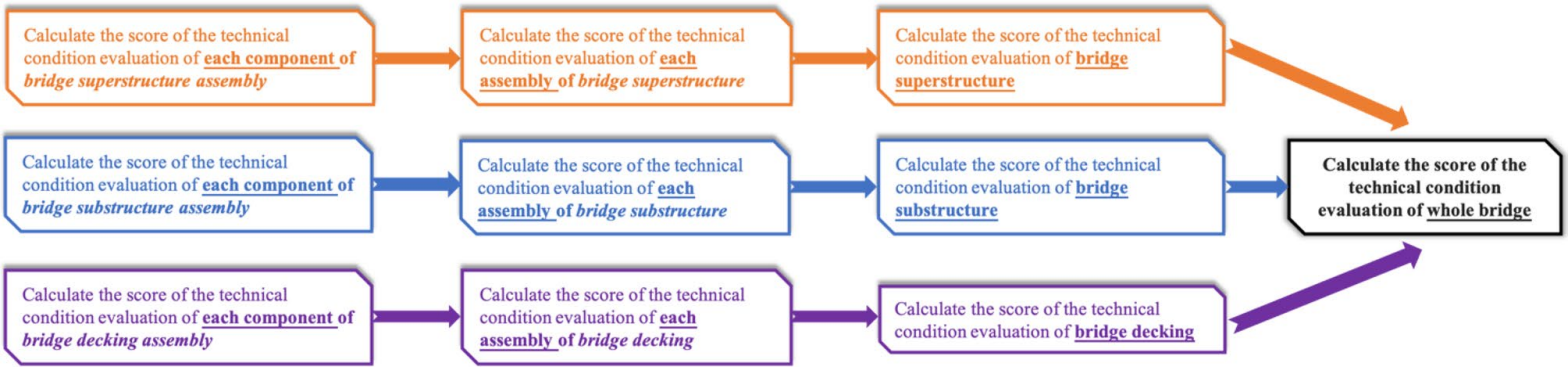
Flow of bridge technical condition evaluation calculation.

The bridge is divided into three parts containing the superstructure, substructure and bridge decking for calculation. With the method of multi-hierarchical comprehensive evaluation, the component score (PMCI\BMCI\DMCI) is first calculated and subtracted from the initial score of 100 according to the qualitative and quantitative degree of the defect. Then, the assembly score (PCCI\BCCI\DCCI) is calculated on the basis of the component score, relating to the number of components contained in the assembly. Additionally, the structural parts’ score (SPCI\SBCI\BDCI) is calculated according to the assembly score and finally integrated into the evaluation score of the technical condition of the whole bridge (Dr) with a proportional weight. The flowchart of the calculation process is shown in Fig. 6.

For example, the score of the component is calculated according to the detailed information using the following formula.

$${\text{PMCI}}_{{\text{i}}} \left( {{\text{BMCI}}_{{\text{i}}} \;{\text{or}}\;{\text{DMCI}}_{{\text{i}}} } \right){ = 100} - \mathop \sum \limits_{{\text{x = 1}}}^{{\text{k}}} {\text{U}}_{{\text{x}}}$$

when x = 1

$${\text{U}}_{{1}} = {\text{DP}}_{{{\text{i1}}}}$$

when x ≥ 2

$${\text{U}}_{{\text{x}}} { = }\frac{{{\text{DP}}_{{{\text{ij}}}} }}{{{{100 \times }}\sqrt {\text{x}} }}{{ \times }}\left( {{100 - }\mathop \sum \limits_{{\text{y = 1}}}^{{\text{x - 1}}} {\text{U}}_{{\text{y}}} } \right){\text{, j}} = {\text{x}}$$

when DP_ij_ = 100

$${\text{PMCI}}_{{\text{i}}} \left( {{\text{BMCI}}_{{\text{i}}} \;{\text{or}}\;{\text{DMCI}}_{{\text{i}}} } \right){ = 0}$$

PMCI_i_-Score of level I and type l of superstructure component, 0–100; BMCI_i_-Score of level i and type 1 of superstructure component, 0–100; DMCI_i_-Score of level i and type 1 of superstructure component, 0–100; k-Number of index types with deductions for level I and type 1 components; ${\text{U}},{\text{x}},{\text{y - Variables;}}$ i-Classification of components, such as 1 for bridge superstructure, 2 for bridge substructure; j-Class j inspections index of level i and type of components; DP_ij_-Deduction scores of the class j inspection index i and type 1 of the component.

During the process of calculation, each component, assembly, structural part and the whole bridge will obtain a score for the convenience of evaluating the technical condition of the bridge separately and entirely. With the visualization and informatization of the BIM function, each segment is displayed in a different colour that provides advanced warning in a bright colour when the score is below a certain range to prevent accidents. In addition, the real-time information of bridge safety conditions is updated for sharing with stakeholders.

### Recommendations for bridge maintenance suggestions

The BMS contains a database of relevant specifications for bridge maintenance and repair to provide corresponding policy recommendations. According to the Bridge Structure Health Grade Assessment in the Technical Specifications for Structural Monitoring of Highway Bridges (JT/T 1037-2022)^27^, the priority orders of maintenance are divided into four levels based on the score of technical condition classification. From level 1 to level 4, the risk of bridge safety gradually increases, the priority of level 1 is a recommendation to solve the defect problems, level 2 suggests paying attention to defect conditions in daily inspection, level 3 means solving the defect threats as soon as possible, and level 4 indicates that the bridge defect needs to be solved urgently or a threat to the safety of a bridge exists. Table 4 shows the relation between bridge defects and the priority of maintenance suggestions. The solved defect problems are marked ‘completed’, and repair records are stored in the system, which enables the management personnel to propose more targeted maintenance options based on historical records.

**Table 4:** Priority maintenance suggestions for bridge defects.

Type of bridge defect	Score of bridge technical condition	Level of bridge defect	Priority of bridge maintenance suggestion
Damaged: surface cracks	Above 80	1	Common: routine maintenance
Corrosion: cable corrosion	60–80	2	Normal: small-scale repair
Deficiency: abutment defect	40–60	3	Rapid: moderate-scale repair
Distortion: pier deformation	Below 40	4	Urgent: large-scale repair

## Case application

### Establishment of the cocodala bridge BIM model

**Figure 7:**
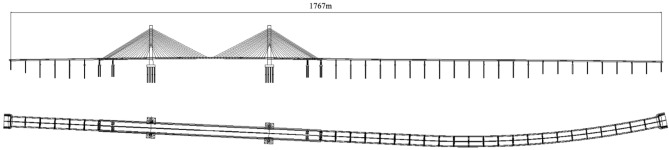
Plane linear drawing of the Cocodala Bridge.

The Cocodala Bridge is 1767 m long across the Ili River, which is in Cocodala City, Xinjiang Uygur Autonomous Region, China. The plane linear drawing of the bridge is located on a straight line (initial pile number K8 + 796.46, termination pile number K9 + 922.69) and a circular curve (initial pile number K9 + 922.69, termination pile number K10 + 563.54, radius 2500 m, left deviation), and the longitudinal section of the bridge is located on a vertical curve of radius 12,000 m (Fig. 7). The main bridge is a prestressed concrete cable-stayed bridge with an H-shaped double-plane tower that is 600 m long and 3.25 m long with a bidirectional slope of 1.5%. The north and south approach bridges are 240 and 920 m long, respectively, 29.5 m wide with a simple-continuous prestressed concrete T-beam.

**Figure 8:**
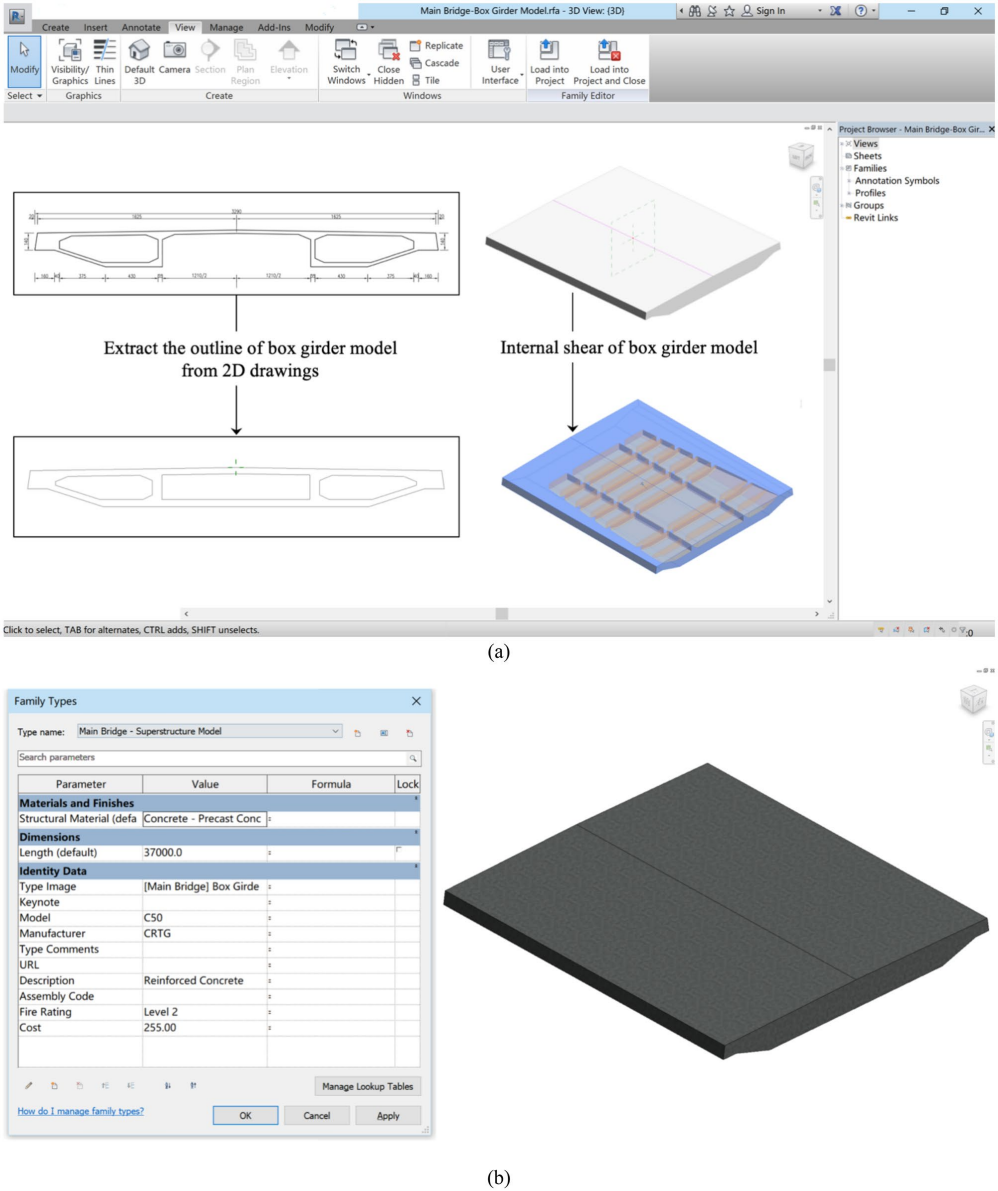
(**a**) Establishment of the BIM3D model of the box girder of the main bridge; (**b**) Properties of the BIM3D model of the box girder of the main bridge. Linear drawing of the Cocodala Bridge.

The real bridge BIM model is imported into the BMS to realize the functions of simulation and analysis, which is the data source of model layer information. A BIM model of the Cocodala Bridge is established using Revit 2017 software with a parameterization method, which can quickly generate different specifications of similar components by changing the dimension parameters. The contour lines in the drawings of components are extracted to create the BIM3D model. Furthermore, the internal structure is sheared to precisely show the real condition of a single component (Fig. 8a). For the whole splicing of the bridge, different segmentation models are established according to the assembled inclination angle. In addition, the actual information of components is added to the properties of the model, such as material types, concrete grade, construction organization and cost, creating a BIM model covering on-site bridge information (Fig. 8b) to facilitate the rapid and centralized search of bridge information during the maintenance process.

**Figure 9:**
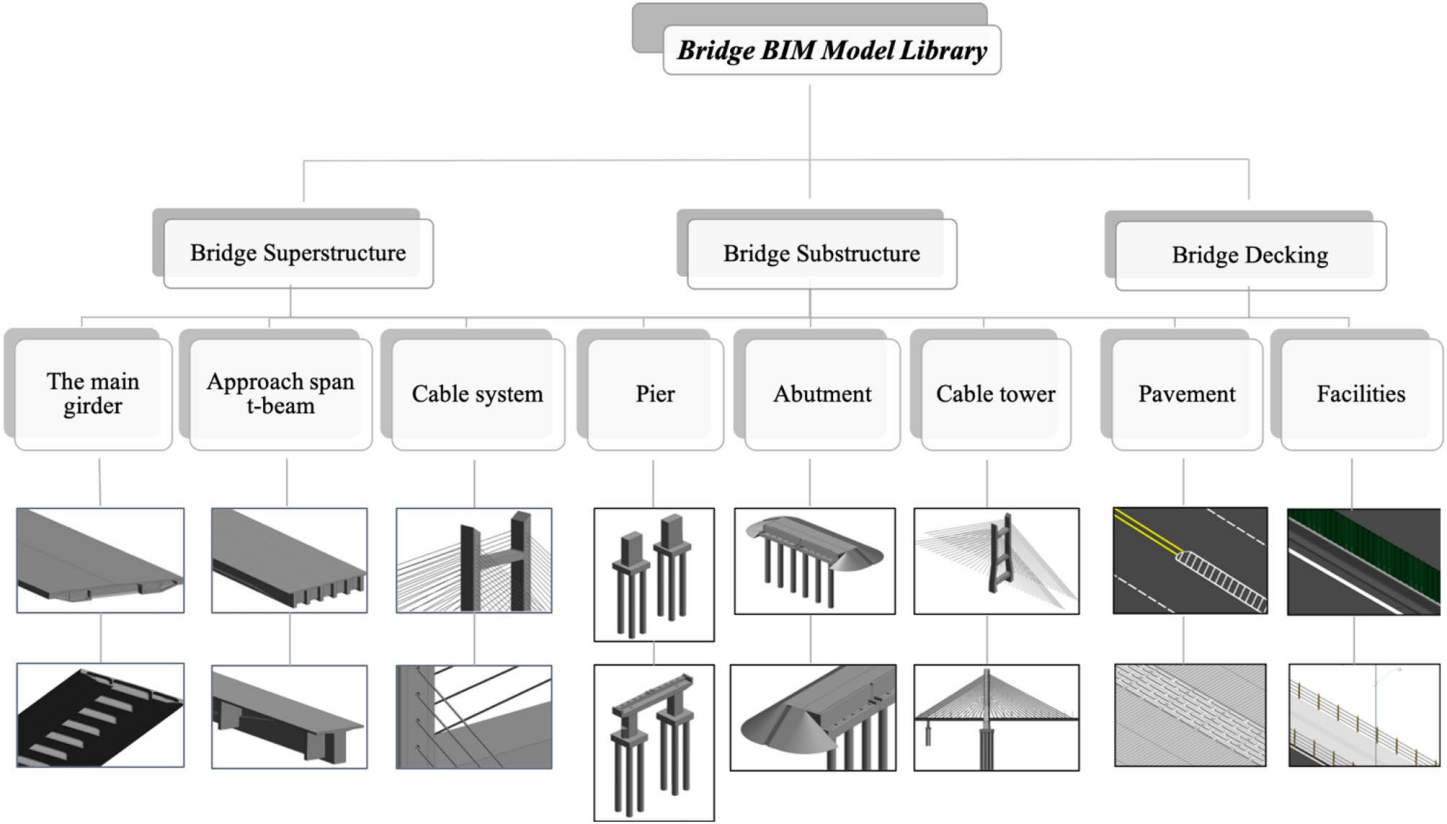
Classifications of the Cocodala Bridge BIM model library.

**Figure 10:**
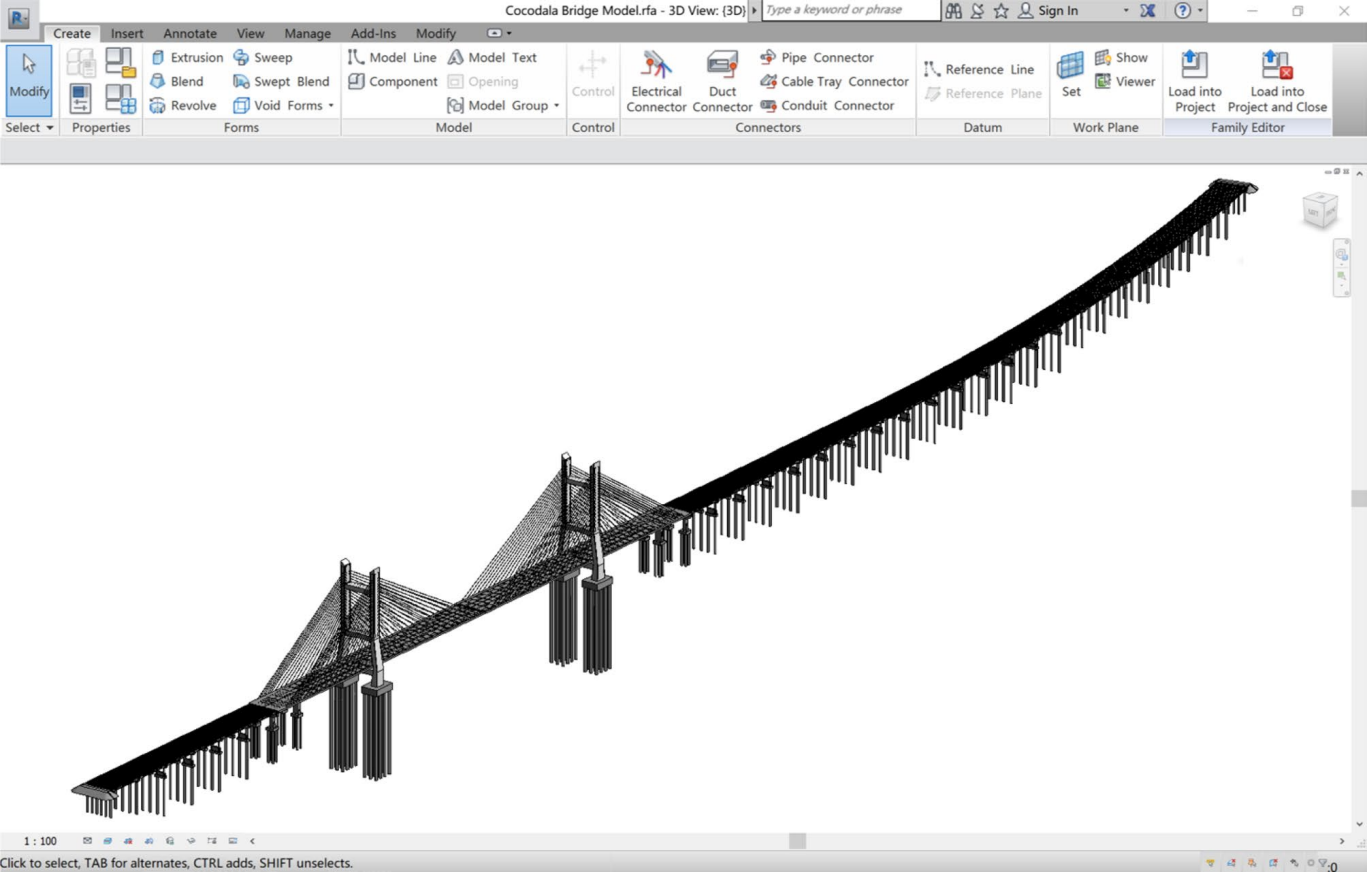
BIM3D model of the Cocodala Bridge.

The bridge BIM model library consists of the superstructure, substructure and bridge decking, the classifications of which are shown in Fig. 9, and the whole bridge is assembled according to the layout and composition (Fig. 10).

**Figure 11:**
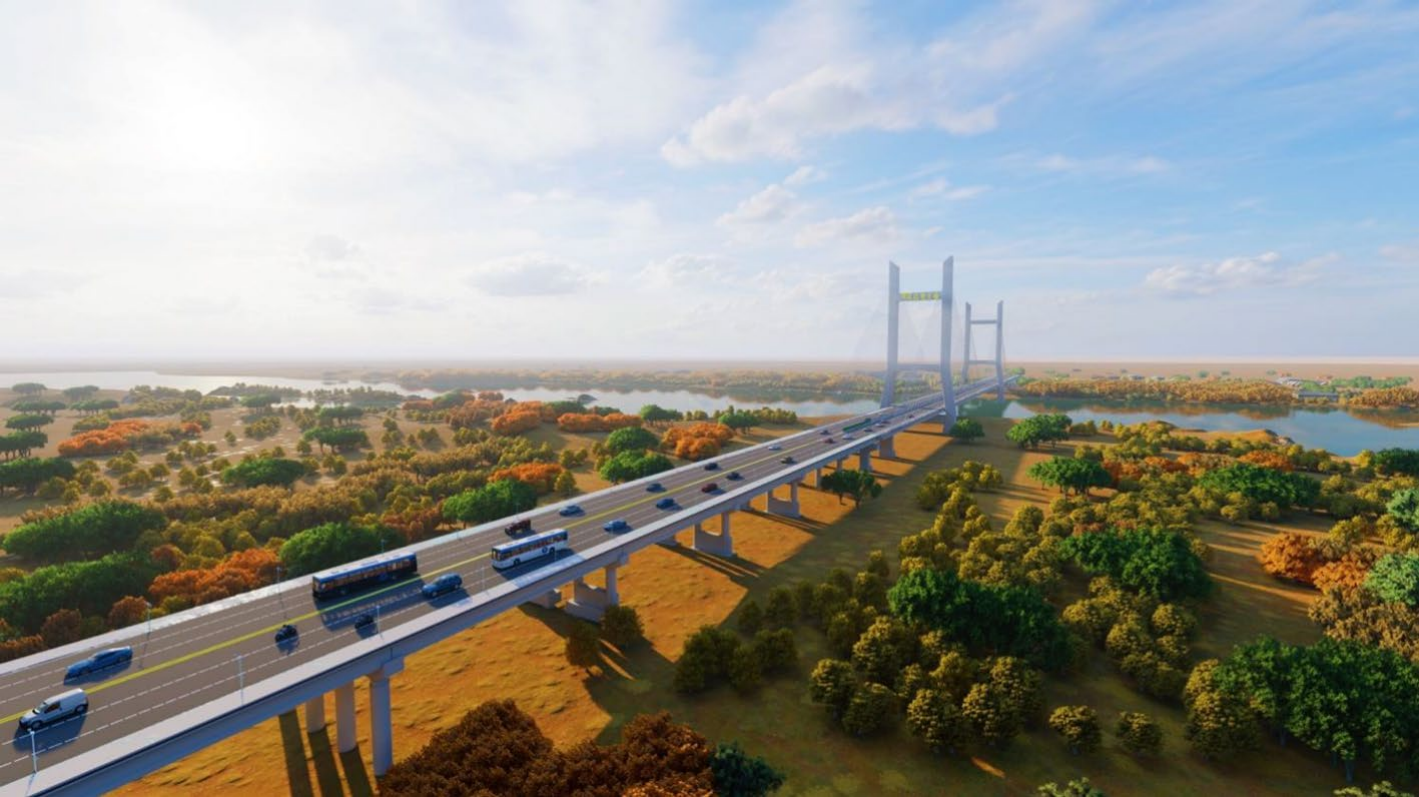
Rendering effect of the Cocodala Bridge BIM model.

The surrounding environment model, such as the river and terrain, is created to establish lighting and rendering (Fig. 11) to truly display the real bridge conditions and realize the visualization function of BIM technology.

### Collection of the cocodala bridge defect data

**Figure 12:**
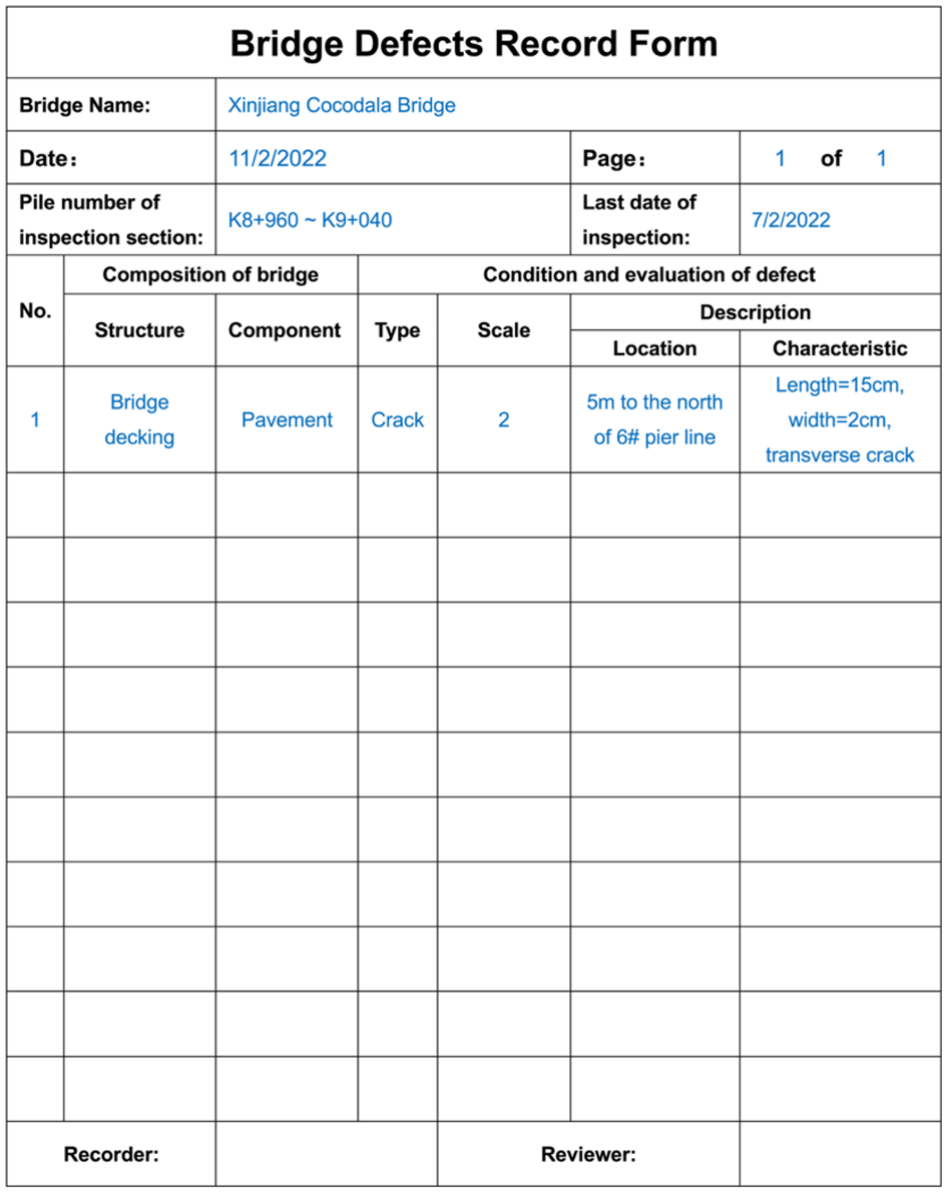
Bridge defect record form for data collection.

To ensure the safe operation of a bridge, regular bridge inspections are carried out to find defect conditions twice a week. The inspector personnel record the defect conditions, including scale, location, and description, in a form (Fig. 12) during bridge inspection and upload them to the system in a timely manner.

**Figure 13:**
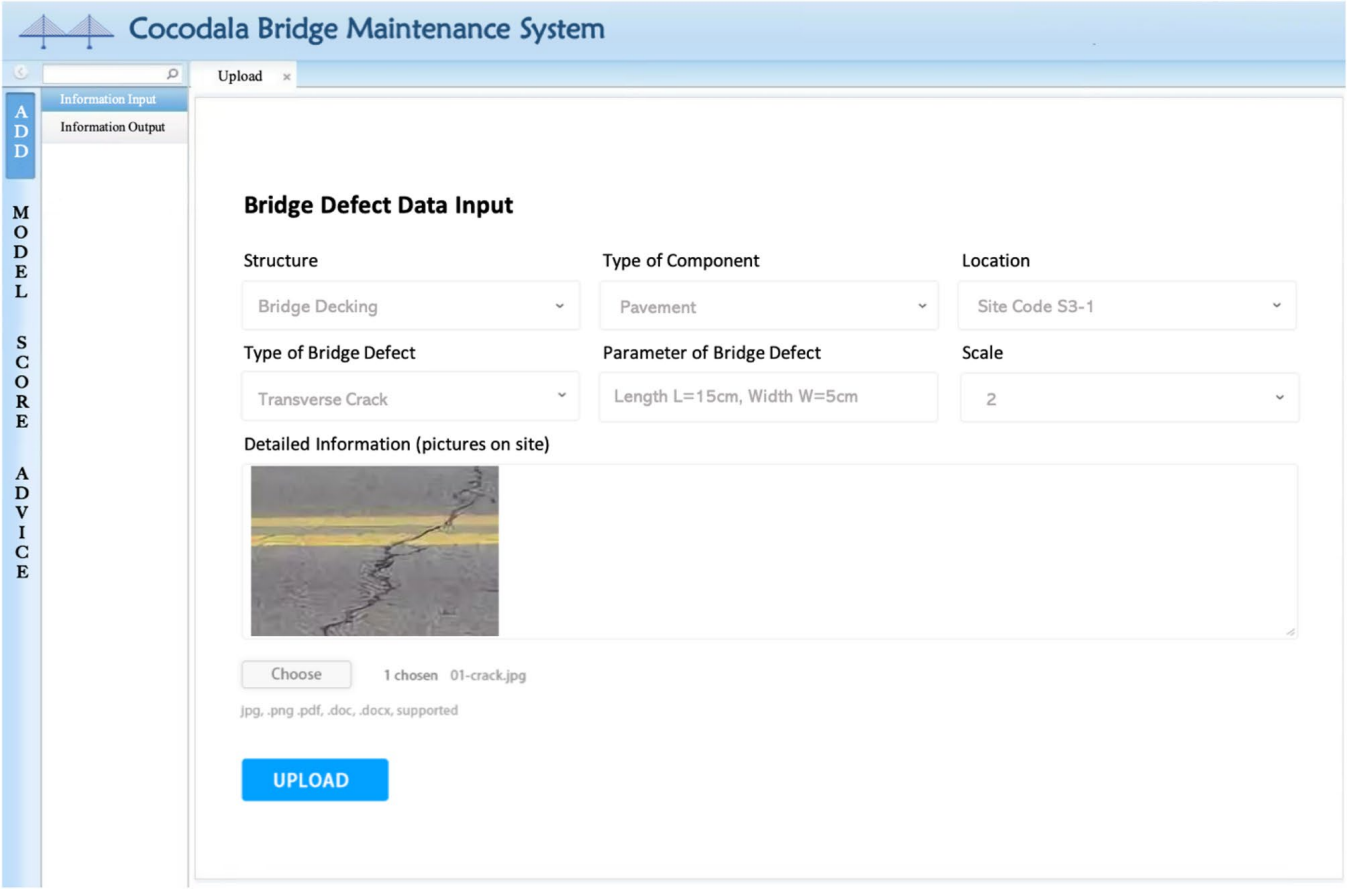
Interface for inputting bridge inspection data into the bridge maintenance system.

According to the form, inspector personnel upload bridge defect data within a unified interface (Fig. 13) in BMS, which standardizes the method of recording defect information and ensures data accuracy. Based on the bridge defect inspection information input, a bridge defect database is established to provide support for the functional utilization of the model and data layers.

### Utilization of the BIM-based cocodala bridge defect maintenance system

**Figure 14:**
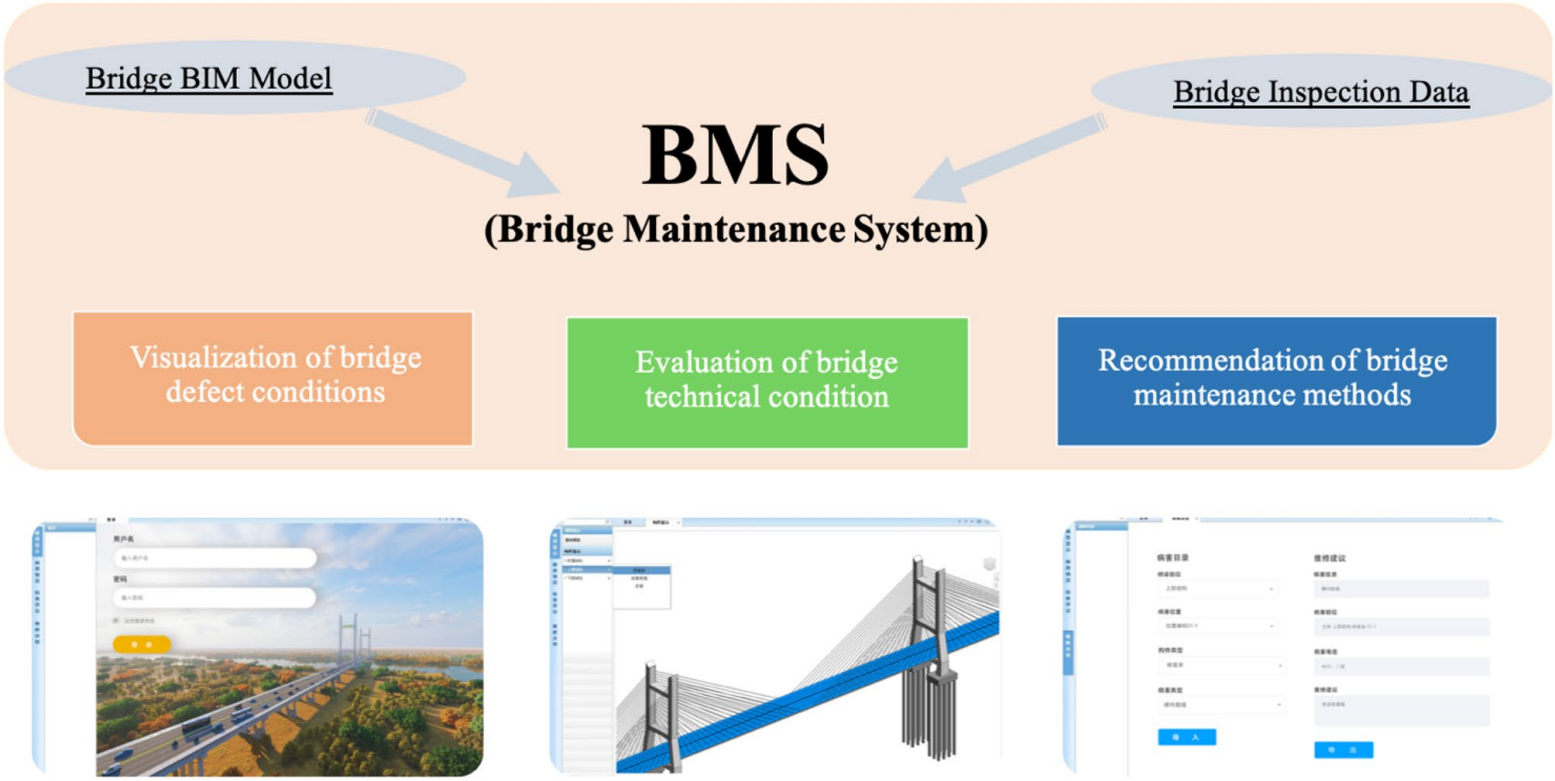
BMS utilization flow.

The bridge BIM model and inspection data are imported into the BMS to realize the functions of visualization, evaluation and recommendation (Fig. 14). The function of ‘visualization of bridge defect conditions’ is realized by combining the bridge model with the defect model, and the bridge defect database is constructed with the inspection data to realize the function of ‘evaluation of bridge technical conditions’ and ‘recommendations of bridge maintenance methods’.

**Figure 15:**
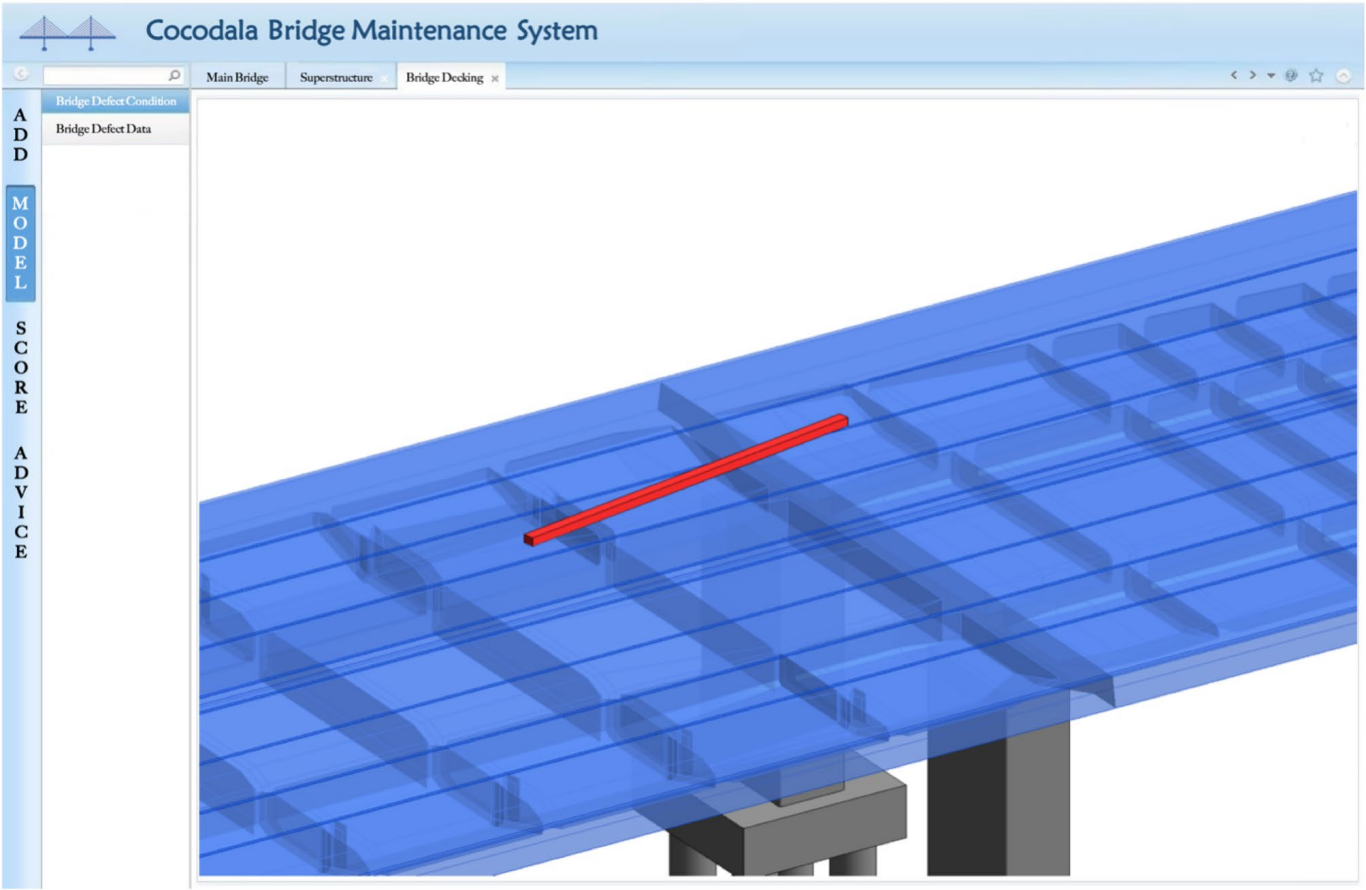
Modelling display of the ‘Deck-cracks’ bridge defect condition on the BMS website.

The function of ‘visualization of bridge defect conditions’ is utilized in the BMS as a basic function of BIM technology. The established bridge BIM model is exported to the IFC model with IFC 2 × 3 Coordination View, version 2.0, which provides the information to the resource layer and then converts it to the object file (OBJ) model containing geometries and positions. Finally, the OBJ model is loaded into the system platform to be combined with the defect model which is transformed in the same way. According to the defect data input, the correlation defect BIM model to add is selected and synthesized to the corresponding location of the bridge BIM model by corresponding the ID attribute in the MySQL database, thus achieving a visualization display of defect conditions on the bridge (Fig. 15).

**Figure 16:**
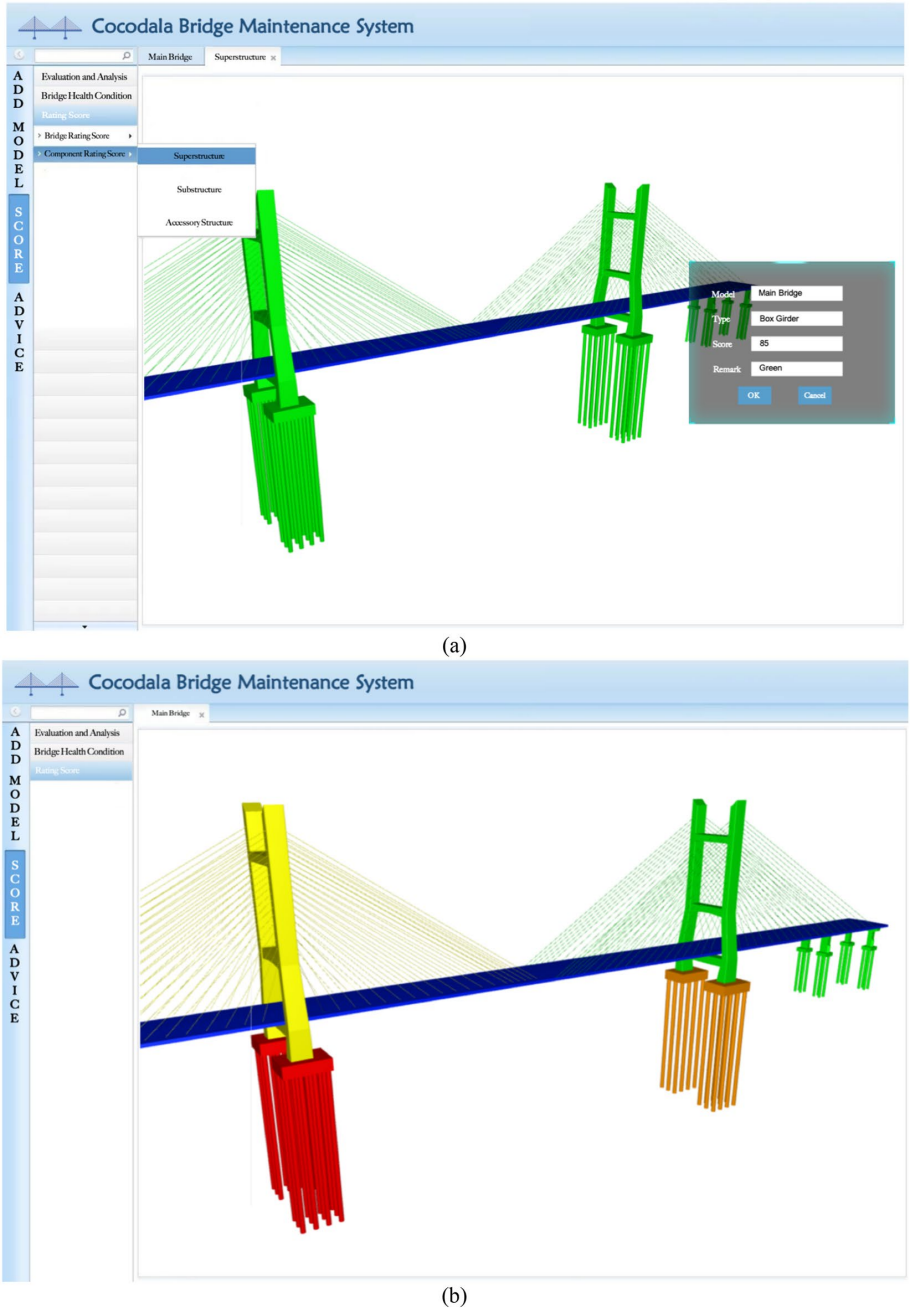
(**a**) Simulation colour display of 85 scores of the box girder in the bridge superstructure; (**b**) Simulation colour display of the overall score of the bridge.

The scores calculated according to the bridge technical condition evaluation formula are displayed on the bridge model in different colours according to the requirements specification in the Standards for Technical Condition Evaluation of Highway Bridges (JTG/T H21-2021)^26^, of which above 95 is blue, between 80 and 95 is green, between 60 and 80 is yellow, between 40 and 60 is orange, and below 40 is red. Figure 16a shows the simulation of 85 scores of the box girder in the superstructure of the bridge, and Fig. 16b shows the simulation of the overall technical condition of the bridge within the range of all scores. Based on the bridge defect database, the comprehensive evaluation of technical conditions can be used to estimate the operation condition of the bridge, which realizes the BIM function of information sharing.

**Figure 17:**
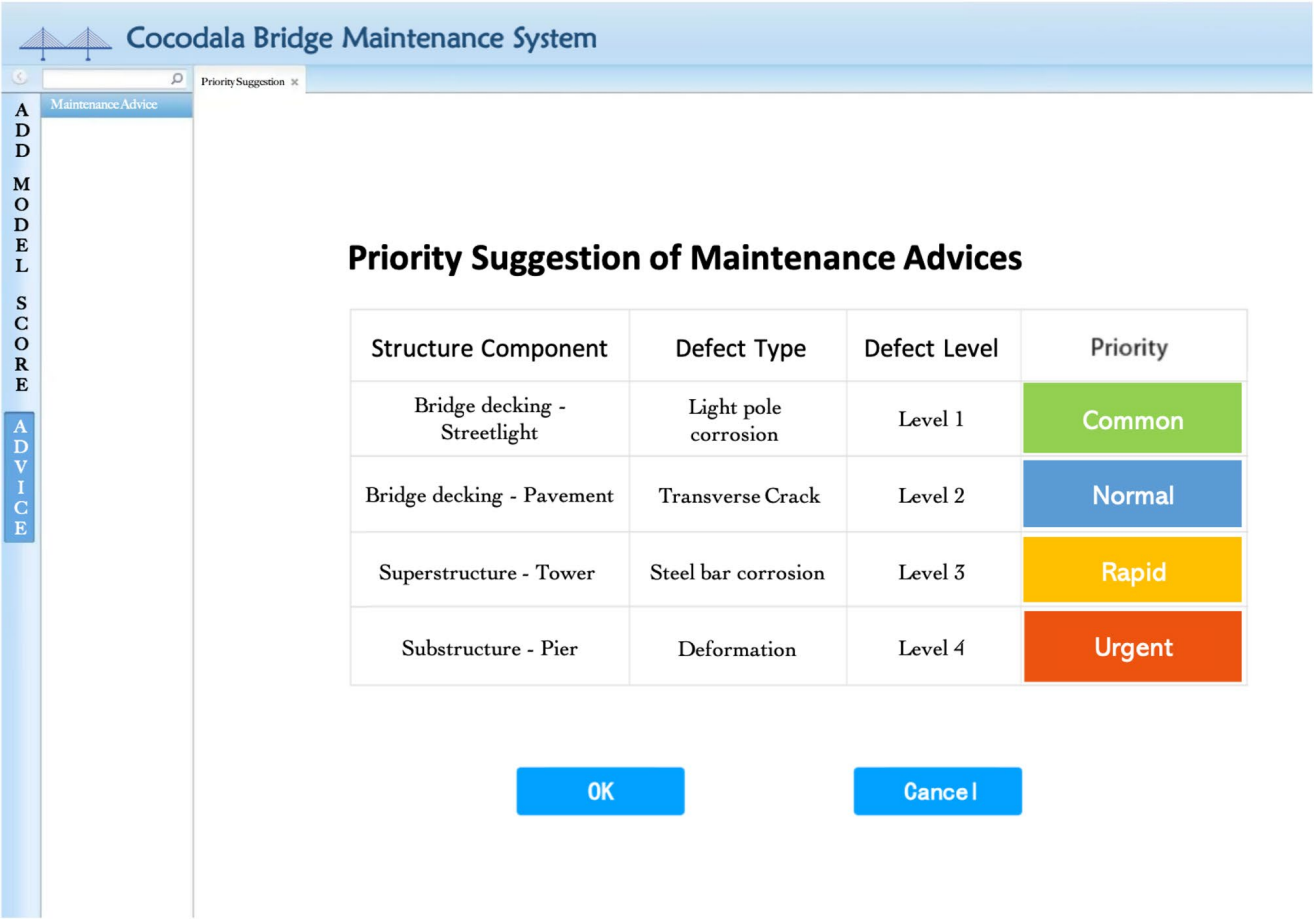
Priority suggestions of bridge maintenance are advised with colour.

In the BMS, existing bridge defects can be viewed, and the priority advice of maintenance suggestions is recommended according to the standard files stored in the system, which realizes the integrated function of BIM technology. As shown in Fig. 17, the green colour indicates that the bridge needs normal routine maintenance, the blue colour indicates small-scale repair, the yellow colour indicates moderate maintenance, and the problems of traffic evacuation during repair should be considered. Finally, the red colour indicates that the bridge has extremely serious damage, which has affected the safe operation of the bridge, and large-scale centralized maintenance must be organized immediately.

## Discussion

The bridge defect maintenance system described in this paper achieves the BIM functions of visualization, informatization and integration and is utilized in all stages of system development. A bridge defect BIM3D model library is established to describe the geometric properties and size information of defects. Bridge inspection data are input into a unified format to encode defect information based on the IFD standard, and then a bridge defect database is established as a basis for the combination of model links and visual display. According to the formula, bridge technical condition scores are displayed in different colours, which is convenient for monitoring bridge operation conditions. In addition, maintenance suggestions are provided by automatically matching the relevant repair method according to the defect type input to realize the digital management utilization of BIM technology. Taking the Cocodala Bridge as a case study to utilize the BMS, a bridge BIM model is established, and inspection data are used to realize the functions of ‘visualization of bridge defect conditions’, ‘evaluation of bridge technical conditions’ and ‘recommendations of bridge maintenance methods’. The defect conditions of the Cocodala Bridge are displayed, and the real-time bridge information is fed back to monitor the operating conditions, achieving the information collaborative sharing function of BIM technology. These functions promote the development of BIM technology based on real engineering data and provide new ideas for the development of bridge maintenance management systems.

The innovation of this paper lies in the function of managing defect data in the BMS, especially concerning defect conditions appearing during bridge inspection and the management of the daily bridge maintenance. The BMS can interactively utilize the relevant functions by importing the bridge BIM model and inspection data, achieving the maintenance of specific bridges. Real-time sharing of bridge operation conditions and defect information statistics can help stakeholders solve problems in a timely manner and ensure the safe operation of bridges.

However, the Revit model is a heavy one with a lot of native information and contained much unnecessary parameters which are removed during the transformation of the Revit model to OBJ model, making the mapping procedure of the IFC export with limitation and inefficiency. In the future plan of improvement, we will consider more about adopting the most appropriate modeling method according to the function requirement and then complete the development of functional modules on the basic model with native information. And for the utilization of BIM technology in bridge maintenance during the primary stage, the overall function cannot be completely developed for a BMS for bridges with existing weaknesses. For the collection process of bridge inspection, we found that the inspection personnel could not record the accurate position of defects on bridges by the code. A kind of quick response (QR) code that contains the information of bridge components must be developed so that the inspection personnel can use a BIM mobile device to scan a QR code attached to bridge components to acquire more accurate locations and information^28^, substituting for manual record methods. The system lacks a structure analysis tool to assess the structural condition of bridges for providing early warning of bridge safety risks as well as monitoring the bridge health condition^29^. At present, structural monitoring sensors have been installed at the key structure of the Cocodala Bridge, collecting data for the analysis of structural behaviour. In the future, we will strive to combine the system with the structure monitoring system to realize more functions of bridge maintenance and enhance its convenience in serving more bridges.

## Conclusion

Through the development and utilization of a BIM-based BMS, the following conclusions can be drawn:

- A BMS is developed based on BIM that realizes the functions of visualization, informatization and integration, and a bridge defect model library is developed;
- A bridge defect database is established based on bridge inspection data, and the defect data are encoded and classified using the IFD standard and displayed through the BIM visualization function;
- The technical condition of a bridge is evaluated and displayed in different colours according to the rating score range in the bridge model;
- Maintenance suggestions and priority advice are matched to the bridge defect according to relevant normative documents and scores of technical condition evaluation;
- Taking the Cocodala Bridge in Xinjiang, China, as a case study, a bridge BIM3D model is established and imported into the BMS to utilize the function of ‘visualization of bridge defect conditions’, and the inspection data are imported synchronously to utilize the function of ‘evaluation of bridge technical conditions’ and ‘recommendations of bridge maintenance’.

In view of defects appearing in the daily inspection of bridges, the utilization of the BIM technology above can effectively assist bridge maintenance work, which facilitates timely identification and solving of the weaknesses in bridges to ensure the safe operation of bridges in a digital way. At present, BIM technology is also combined with advanced technologies such as sensors and the Internet of Things (IoT), all of which will promote the digitization process of bridge maintenance, making it more convenient and efficient, ultimately realizing the full life cycle management of bridge engineering.

## Data Availability

All data generated or analysed during this study are included in this published article.
